# Perioperative Selective Serotonin Reuptake Inhibitor (SSRI) Use and Postoperative Fracture Fixation Outcomes: A Multi-institutional Retrospective Cohort Study

**DOI:** 10.7759/cureus.114976

**Published:** 2026-08-22

**Authors:** Anish Ponna, Shreyas Soma, Zachary J Demetriou, Alec Giakas, Asif M Ilyas

**Affiliations:** 1 Orthopaedic Surgery, Drexel University, Philadelphia, USA; 2 Orthopaedic Surgery, Rothman Orthopaedics, Philadelphia, USA; 3 Orthopaedic Surgery, Ponce Health Sciences University, Ponce, PRI

**Keywords:** femoral shaft fractures, humeral intramedullary nail, humeral shaft fracture, intramedullary femoral nail, intramedullary nailing of the tibia, mid-shaft clavicular fracture, open reduction internal fixation, selective serotonin reuptake inhibitor, tibial shaft fracture, ulnar shaft fracture

## Abstract

Introduction: Selective serotonin reuptake inhibitors (SSRIs) have been implicated in impaired bone metabolism and delayed fracture healing. This study evaluates the association between perioperative SSRI use and complications following open reduction and internal fixation (ORIF) and intramedullary nailing (IMN) of various long bone shaft fractures. The study hypothesis is that perioperative SSRI use negatively affects fracture healing and increases adverse postoperative outcomes across fixation constructs.

Methods: The TriNetX database was used to identify patients (≥18 years) undergoing humeral, clavicular, or ulna shaft ORIF and humeral, femoral, or tibial shaft IMN. Patients were stratified by SSRI use within one month of the index surgery; controls had no documented SSRI history. Propensity score matching controlled for demographics and relevant comorbidities. Outcomes were assessed at one-year after surgery. Measures of association, including risk ratios (RRs), 95% confidence intervals (CIs), and p-values, were used to compare outcomes between cohorts.

Results: In the humeral shaft ORIF cohort (n=1,576), SSRI exposure was associated with significantly higher rates of nonunion (RR=1.51, 95% CI: 1.14-2.01; p=0.004) and infection-related implant failure (RR=2.03, 95% CI: 1.31-3.16; p=0.001). In the ulna shaft ORIF cohort (n=567), SSRI use was associated with increased infection-related implant failure (RR=1.94, 95% CI: 1.12-3.39; p=0.017) and mechanical implant failure (RR=1.91, 95% CI: 1.14-3.19; p=0.012). No significant associations were identified in the clavicle shaft ORIF cohort or in any IMN cohort (p>0.05).

Conclusion: The study hypothesis was not upheld. While significant adverse associations were observed in select ORIF cohorts, there were no significant differences in adverse outcomes in IMN cohorts. This discrepancy limits the ability to draw an association between SSRI use and impaired fracture healing. Given the higher baseline nonunion rates associated with IMN, the observed differences between cohorts appear inconsistent and likely reflect confounding rather than a true effect of SSRI use.

## Introduction

Selective serotonin reuptake inhibitors (SSRIs) are one of the most widely prescribed drug classes in the United States, serving as first-line pharmacotherapy for several conditions, including major depressive disorder (MDD), generalized anxiety disorder, panic disorder, and more [1-3]. SSRIs exert their effects by selectively blocking the serotonin transporter at the presynaptic neuron, increasing serotonin concentrations within the synaptic cleft, and enhancing serotonergic signaling [4]. Their favorable safety profile has contributed to widespread, long-term use across diverse patient populations, including those presenting for elective and urgent surgical care [2].

Beyond their central neuropsychiatric effects, SSRIs exert significant influence on skeletal homeostasis through both direct peripheral and indirect central mechanisms. Peripherally, osteoblasts and osteoclasts express several receptors and enzymes that regulate bone cell differentiation, proliferation, and apoptosis [5]. Additionally, osteoclasts demonstrate elevated native expression of the direct molecular target of SSRIs, rendering bone-resorptive cells particularly susceptible to serotonergic modulation [5-7]. Centrally, chronic SSRI use drives a serotonin-dependent increase in sympathetic nervous system output that activates the receptor activator of nuclear factor kappa-B ligand pathway, further stimulating osteoclastogenesis while suppressing osteoblast activity [8]. Together, these mechanisms yield a well-characterized pattern of skeletal decline in SSRI-exposed patients, including reduced bone mineral density, cortical bone deterioration, elevated fracture risk, and higher rates of osteoporosis [9-13].

Despite this established mechanistic and epidemiological framework, the clinical implications of perioperative SSRI exposure in the context of acute fracture surgery remain poorly characterized. Open reduction and internal fixation (ORIF) and intramedullary nailing (IMN) are among the most commonly employed operative techniques for long bone fracture stabilization [14]. ORIF achieves rigid fixation that promotes primary cortical healing via direct intramembranous ossification [15], whereas IMN provides relative stability that promotes secondary healing via endochondral callus formation [16]. Both pathways depend on coordinated biological cascades, including inflammation, osteoblast-mediated bone formation, and remodeling, each of which has been shown to be susceptible to disruption by serotonergic modulation [17]. Given the mechanistic evidence linking SSRIs to bone metabolism pathways, biological plausibility exists for an adverse effect of perioperative SSRI use on postoperative fracture healing. However, large-scale, multi-institutional data examining this relationship are limited in the current literature. Therefore, this study aims to evaluate the association between perioperative SSRI use and rates of nonunion, infection-related implant failure, mechanical implant failure, and all-cause mortality at one year following ORIF and IMN fixation of long bone fractures.

## Materials and methods

Study design

This study was conducted as a multi-institutional retrospective cohort study using a large, federated electronic health record (EHR) database. The study adhered to the Strengthening the Reporting of Observational Studies in Epidemiology (STROBE) guidelines for observational research. Institutional review board approval was not required given the de-identified and aggregated nature of the data.

Database

Patient data were obtained from the TriNetX Research Network. TriNetX is a global research network encompassing data from over 170 healthcare organizations and more than 400 million patients. It contains de-identified aggregate patient information covering procedures, diagnoses, medications, vitals, genomics, and demographics. Healthcare organizations involved in the TriNetX network contribute healthcare data in de-identified, pseudo-anonymized, or limited data set formats, following local privacy regulations. The de-identification process conforms to the HIPAA Privacy Rule standards, as verified by a qualified expert, and meets the requirements of Section §164.514(b)(1), ensuring HIPAA compliance.

Patient population

Patients were identified using International Classification of Diseases, 10th revision (ICD-10) diagnosis codes and Current Procedural Terminology (CPT) codes corresponding to fracture fixation surgeries of interest. Six cohorts were constructed: three ORIF (humeral, clavicular, and ulnar shaft fractures) and three IMN (humeral, femoral, and tibial shaft fractures). Each cohort was analyzed independently (Appendices A, B).

Inclusion criteria were: (1) age ≥18 years at the time of index surgery, (2) a qualifying fracture diagnosis, and (3) documentation of the relevant fixation surgery within the study period. Patients were excluded if they had: (1) a prior history of the same or ipsilateral fracture surgery, (2) polytrauma as the primary admission diagnosis, (3) metabolic bone disease, (4) active malignancy involving bone, (5) prior bisphosphonate or teriparatide use, or (6) insufficient follow-up data within one year of the index procedure.

Cohort stratification

Within each of the six cohorts, patients were stratified into two groups based on perioperative SSRI use. The SSRI cohort consisted of patients with any documented use of SSRIs, including citalopram, escitalopram, fluoxetine, paroxetine, or sertraline, within one month before or following the index surgery (Appendix C). The control group consisted of patients with no documented history of SSRI used in their EHR.

Propensity score matching

To reduce confounding by indication and baseline demographic characteristics, cohorts were propensity score-matched using a 1:1 greedy nearest neighbor algorithm with a caliper of 0.1 pooled standard deviations. Covariates included in the model were age, sex, BMI, smoking status, and several medical comorbidities. Standardized mean differences (SMDs) were calculated before and after matching; an SMD of <0.10 was considered indicative of adequate covariate balance.

Outcomes

Outcomes were assessed at one-year following the index procedure. Primary outcomes included: (1) nonunion, (2) infection-related implant failure, (3) mechanical implant failure, and (4) all-cause mortality (Appendix D).

Statistical analysis

Descriptive statistics were reported for all baseline characteristics before and after propensity score matching. For each outcome, measures of association were calculated, including risk ratios (RRs) with 95% confidence intervals (CIs) and corresponding p-values. Statistical significance was defined as p<0.05. All analyses were performed using the built-in analytical tools within the TriNetX platform.

## Results

Cohort characteristics

Following matching, six independent cohorts were established: humeral shaft ORIF (n=1,576 per group), clavicle shaft ORIF (n=813 per group), ulna shaft ORIF (n=567 per group), humeral shaft IMN (n=485 per group), femoral shaft IMN (n=3,005 per group), and tibial shaft IMN (n=3,377 per group). After matching, all cohorts demonstrated covariate balance across baseline demographic and comorbidities (Tables 1, 2).

**Table 1:** Baseline characteristics after propensity score matching: ORIF cohorts Values shown as % of cohort for categorical variables and mean ± SD for continuous variables. SMD<0.10 indicates adequate balance. Propensity score matching was performed using a 1:1 nearest-neighbor algorithm without replacement. BMI, body mass index; COPD, chronic obstructive pulmonary disease; Ctrl, control; ORIF, open reduction and internal fixation; SD, standard deviation; SMD, standardized mean difference; SSRI, selective serotonin reuptake inhibitor.

	Humeral ORIF (n=1,576)			Clavicle ORIF (n=813)			Ulna ORIF (n=567)		
	SSRI	Ctrl	SMD	SSRI	Ctrl	SMD	SSRI	Ctrl	SMD
Demographics									
Age (mean ± SD)	56.8±19.6	57.8±19.9	0.053	42.4±16.7	43.4±16.8	0.056	51.4±19.0	51.4±19.7	0.002
Female (%)	71.0	71.1	0.001	40.7	43.8	0.062	62.4	66.1	0.077
White (%)	84.5	85.8	0.037	87.8	89.1	0.038	84.0	87.1	0.090
Black (%)	8.1	7.4	0.026	4.7	4.1	0.030	10.6	8.8	0.060
Hispanic (%)	4.7	4.2	0.025	4.6	3.9	0.031	5.6	5.8	0.008
BMI (mean ± SD)	31.1±8.2	30.7±7.7	0.051	27.3±6.3	27.0±6.1	0.060	28.7±7.2	27.9±6.7	0.120
Comorbidities									
Diabetes mellitus (%)	25.9	24.4	0.035	8.9	9.3	0.017	16.0	14.6	0.039
Hypertension (%)	56.3	57.3	0.020	32.3	30.8	0.034	48.1	45.3	0.057
Heart failure (%)	11.9	11.9	<0.001	2.5	2.3	0.008	7.1	6.2	0.035
COPD (%)	12.5	10.9	0.049	5.3	4.6	0.034	10.9	9.2	0.059
Chronic kidney disease (%)	12.2	10.9	0.040	3.0	3.0	<0.001	6.9	7.1	0.007
Opioid-related disorders (%)	4.5	4.3	0.012	4.9	3.3	0.081	4.9	5.6	0.032
Osteoporosis (%)	16.5	16.0	0.013	5.3	5.8	0.024	11.6	12.0	0.018
Nicotine dependence (%)	23.9	24.3	0.010	26.9	27.1	0.003	30.5	31.2	0.015
Alcohol use disorder (%)	12.8	13.4	0.019	15.0	13.8	0.035	15.9	14.6	0.034
Vitamin D deficiency (%)	16.0	15.9	0.003	8.4	8.1	0.009	14.1	13.1	0.031
Medications									
Corticosteroids (%)	56.1	55.1	0.020	45.3	46.0	0.015	52.4	52.9	0.011
Proton pump inhibitors (%)	45.4	44.0	0.028	34.1	32.8	0.026	40.7	38.4	0.047

**Table 2:** Baseline characteristics after propensity score matching: IMN cohorts Values shown as % of cohort for categorical variables and mean ± SD for continuous variables. SMD<0.10 indicates adequate balance. "—" indicates variable not included in the matching model for that cohort. Propensity score matching was performed using a 1:1 nearest-neighbor algorithm without replacement. COPD, chronic obstructive pulmonary disease; Ctrl, control; IMN, intramedullary nailing; SD, standard deviation; SMD, standardized mean difference; SSRI, selective serotonin reuptake inhibitor.

	Humeral IMN (n=485)			Femoral IMN (n=3,005)			Tibial IMN (n=3,377)		
										SSRI	Ctrl	SMD	SSRI	Ctrl	SMD	SSRI	Ctrl	SMD
Demographics									
Age (mean ± SD)	62.8±17.1	64.2±17.4	0.083	55.6±23.4	56.6±23.6	0.045	49.7±18.2	50.7±18.7	0.055
Female (%)	70.1	71.5	0.032	65.0	64.5	0.012	54.6	54.2	0.008
White (%)	83.1	85.4	0.062	80.7	81.7	0.027	82.1	82.4	0.009
Black (%)	7.6	6.2	0.057	12.2	11.3	0.029	10.5	9.9	0.018
Hispanic (%)	6.2	7.6	0.057	4.3	4.1	0.010	5.2	4.9	0.012
BMI (mean ± SD)	29.7±8.1	29.6±7.9	0.010	30.0±8.5	29.2±8.2	0.096	29.6±7.2	29.3±7.1	0.043
Comorbidities									
Diabetes mellitus (%)	30.9	29.3	0.036	24.2	23.8	0.009	22.1	21.4	0.017
Hypertension (%)	65.2	64.9	0.004	53.2	53.4	0.005	46.6	46.8	0.005
Heart failure (%)	15.3	16.3	0.028	17.3	16.0	0.034	10.2	9.3	0.032
COPD (%)	16.7	15.5	0.034	13.1	12.1	0.030	12.7	11.4	0.040
Chronic kidney disease (%)	17.3	17.5	0.005	15.6	14.7	0.025	11.2	10.5	0.022
Opioid-related disorders (%)	3.7	3.3	0.022	5.2	5.3	0.006	7.7	7.1	0.020
Osteoporosis (%)	—	—	—	27.2	25.2	0.046	12.3	11.1	0.038
Nicotine dependence (%)	—	—	—	23.4	23.3	0.004	32.7	32.0	0.015
Alcohol use disorder (%)	—	—	—	10.4	10.3	0.003	17.9	16.9	0.026
Vitamin D deficiency (%)	—	—	—	16.0	15.2	0.021	15.6	14.8	0.021
Medications									
Corticosteroids (%)	—	—	—	54.9	55.0	0.001	55.3	57.0	0.034
Proton pump inhibitors (%)	—	—	—	46.6	45.7	0.018	40.7	38.5	0.044

ORIF cohorts

In the humeral shaft ORIF cohort, perioperative SSRI use was associated with significantly higher risk of nonunion (7.1% vs. 4.7%; RR=1.51, 95% CI: 1.14-2.01; p=0.004) and infection-related implant failure (3.7% vs. 1.8%; RR=2.03, 95% CI: 1.31-3.16; p=0.001) than controls. Mechanical implant failure (4.9% vs. 3.5%; RR=1.40, 95% CI: 1.00-1.97; p=0.050) trended toward a higher risk in the SSRI group but did not reach significant levels. No significant differences were found in all-cause mortality (3.7% vs. 4.4%; RR=0.84, 95% CI: 0.60-1.18; p=0.323) (Table 3).

**Table 3:** One-year postoperative outcomes by SSRI exposure: ORIF cohorts Risk ratios (RRs) with 95% confidence intervals (CIs) and p-values comparing SSRI vs. control cohorts at one year following the index procedure. Bolded p-values indicate statistical significance (p<0.05). All comparisons were performed at one year post-index surgery on propensity-matched cohorts. RR>1.0 indicates a higher event rate in the SSRI cohort. "—" indicates outcome not observed or CI not estimable due to zero cell counts. Ctrl, control; ORIF, open reduction and internal fixation; SSRI, selective serotonin reuptake inhibitor.

	Humeral ORIF (n=1,576)				Clavicle ORIF (n=813)				Ulna ORIF (n=567)			
Outcome	SSRI (%)	Ctrl (%)	RR (95% CI)	p-value	SSRI (%)	Ctrl (%)	RR (95% CI)	p-value	SSRI (%)	Ctrl (%)	RR (95% CI)	p-value
Nonunion	7.1	4.7	1.51 (1.14-2.01)	0.004	2.1	3	0.71 (0.38-1.31)	0.268	3.2	4.1	0.78 (0.43-1.43)	0.426
Infection-related implant failure	3.7	1.8	2.03 (1.31-3.16)	0.001	2.1	1.8	1.13 (0.57-2.25)	0.721	6.2	3.2	1.94 (1.12-3.39)	0.017
Mechanical implant failure	4.9	3.5	1.40 (1.00-1.97)	0.050	3.6	3.3	1.07 (0.64-1.80)	0.786	7.1	3.7	1.91 (1.14-3.19)	0.012
All-cause mortality	3.7	4.4	0.84 (0.60-1.18)	0.323	—	—	—	—	—	—	—	—

In the clavicular shaft ORIF cohort, no statistically significant differences were observed between the SSRI and control groups across nonunion (2.1% vs. 3.0%; RR=0.71, p=0.268), infection-related implant failure (2.1% vs. 1.8%; RR=1.13, p=0.721), and mechanical implant failure (3.6% vs. 3.3%; RR=1.07, p=0.786) (Table 3).

In the ulnar shaft ORIF, SSRI exposure was associated with significantly increased risk of infection-related implant failure (6.2% vs. 3.2%; RR=1.94, 95% CI: 1.12-3.39; p=0.017) and mechanical implant failure (7.1% vs. 3.7%; RR=1.91, 95% CI: 1.14-3.19; p=0.012). Nonunion risk did not differ significantly between groups (3.2% vs. 4.1%; RR=0.78, 95% CI: 0.43-1.43; p=0.426) (Table 3).

IMN cohorts

No significant differences in risk were identified across all three IMN cohorts. In the humeral shaft IMN cohort, rates of nonunion (6.0% vs. 7.8%; RR=0.76, p=0.255) and mechanical implant failure (4.1% vs. 2.7%; RR=1.54, p=0.215) were comparable between groups; infection-related implant failure was not observed in either group. In the femoral shaft IMN cohort, nonunion (4.1% vs. 4.1%; RR=1.01, p=0.948), infection-related implant failure (3.1% vs. 2.9%; RR=1.05, p=0.762), and mechanical implant failure (5.1% vs. 4.2%; RR=1.21, p=0.098) were all similar between SSRI and control patients. Likewise, in the tibial shaft IMN cohort, no significant differences were found in nonunion (7.1% vs. 6.3%; RR=1.12, p=0.206), infection-related implant failure (4.9% vs. 5.2%; RR=0.94, p=0.542), or mechanical implant failure (3.8% vs. 3.6%; RR=1.05, p=0.699) (Table 4).

**Table 4:** One-year postoperative outcomes by SSRI exposure: IMN cohorts Risk ratios (RRs) with 95% confidence intervals (CIs) and p-values comparing SSRI vs. control cohorts at one year following the index procedure. All comparisons performed at one-year post-index procedure on propensity-matched cohorts. RR>1.0 indicates a higher event rate in the SSRI cohort. "—" indicates outcome not observed or CI not estimable due to zero cell counts. Ctrl, control; IMN, intramedullary nailing; SSRI, selective serotonin reuptake inhibitor.

	Humeral IMN (n=485)				Femoral IMN (n=3,005)				Tibial IMN (n=3,377)			
Outcome	SSRI (%)	Ctrl (%)	RR (95% CI)	p-value	SSRI (%)	Ctrl (%)	RR (95% CI)	p-value	SSRI (%)	Ctrl (%)	RR (95% CI)	p-value
Nonunion	6	7.8	0.76 (—)	0.255	4.1	4.1	1.01 (0.79-1.29)	0.948	7.1	6.3	1.12 (0.94-1.34)	0.206
Infection-related implant failure	0	0	—	—	3.1	2.9	1.05 (0.78-1.39)	0.762	4.9	5.2	0.94 (0.76-1.15)	0.542
Mechanical implant failure	4.1	2.7	1.54 (—)	0.215	5.1	4.2	1.21 (0.96-1.53)	0.098	3.8	3.6	1.05 (0.82-1.34)	0.699
All-cause mortality	—	—	—	—	7.6	8.1	0.94 (0.79-1.12)	0.472	4.1	4.3	0.95 (0.76-1.20)	0.672

## Discussion

The purpose of this multi-institutional retrospective cohort study was to evaluate the association between perioperative SSRI use and postoperative outcomes in ORIF and IMN fixation techniques of long bones. It was hypothesized that SSRI use would impair fracture healing and lead to increased adverse outcomes. The results showed that in some ORIF cohorts, there were significant associations in increased risk for some fracture patterns; however, some ORIF and all IMN cohorts showed no significant increased risk in poor outcomes. Therefore, the study hypothesis was not upheld. The inconsistency of findings across anatomical sites and fixation methods limits the ability to draw definitive conclusions regarding a true biological effect of SSRIs on fracture healing.

ORIF cohort findings

The significant associations observed in the humeral and ulnar shaft ORIF cohorts are biologically plausible and consistent with the known mechanisms by which SSRIs interfere with bone metabolism. The following mechanistic explanations are offered as biologically plausible hypotheses consistent with the observed associations; they were not directly tested by the present retrospective database analysis. Serotonergic signaling has been shown to disrupt the coordinated osteoclast/osteoblast-mediated remodeling that drives cortical bone healing in ORIF-managed shaft fractures [16,18]. Given that both osteoclasts and osteoblasts express serotonin receptors, perioperative SSRI exposure may impair the local cellular environment necessary for primary cortical healing, thereby increasing susceptibility to nonunion and implant failure.

The humerus and ulna shafts share several anatomic and biomechanical characteristics that may render them vulnerable to potentially SSRI-mediated effects. Both bones are subject to multiplanar loading forces that are transmitted through the upper extremity and rely on robust cortical contact and rigid fixation to achieve primary bone healing [19,20]. The humeral shaft is notable for its dependence on a single nutrient artery entering the middle third, which is vulnerable to disruption by fracture or surgical dissection [21]. When this endosteal supply is compromised, healing becomes dependent on the periosteal circulation and its associated osteoprogenitor cells, both of which may be susceptible to potential SSRI-mediated impairment of osteoblast function and overall bone healing [18,22,23]. Infection-related implant failure, which was observed in both cohorts, may reflect potential SSRI-mediated platelet dysfunction, leading to impaired hemostasis and hematoma formation, a well-established risk factor for wound breakdown and secondary infection [24-26].

The absence of significant findings in the clavicle ORIF cohort may be attributed to multiple factors. The clavicle's midshaft blood supply is entirely periosteal with anastomoses [27]. This redundancy can explain that even if one source was compromised by potential SSRI-mediated bone impairment, the others can compensate. Furthermore, the clavicle is non-weightbearing and clavicle fractures disproportionately affect younger, healthier patients [28]. As a result, surgical intervention of ORIF has been shown to have a near-zero nonunion rate [29]. While SSRIs may still have a negative effect on fracture healing, the factors discussed above may outpower the drug's effects and show no clinical impairment.

IMN cohort findings

In the IMN cohorts, no significant difference in risk was observed for all three fractures. Femoral and tibial shaft fractures managed with IMN are associated with higher baseline nonunion rates than ORIF constructs, and the biological rationale for SSRI interference with bone healing would predict a detectable effect in the large cohorts [30,31]. The paradoxical findings are further refuted by previous literature, which found that IMN, which promotes secondary (endochondral) bone healing via callus formation, may be more vulnerable to poorer SSRI-related healing outcomes [18,32]. The results of this study showing no impairment of bone healing in these IMN cohorts could have been due to confounding variables that were not accounted for during propensity score matching.

More broadly, the inconsistency between significant ORIF and null IMN findings across cohorts with varying sample sizes and baseline risk profiles weakens the case for a uniform causal effect of perioperative SSRI use on fracture fixation outcomes. Residual confounding remains a significant concern even after propensity matching, and the observed associations in the ORIF cohorts may partly reflect unmeasured differences between SSRI users and non-users that the available covariates did not fully capture.

Comparison to existing literature

The findings of this study fit into a growing body of evidence linking SSRI use to adverse skeletal outcomes. Prior epidemiological studies have demonstrated associations between chronic SSRI use and reduced bone mineral density, increased cortical bone loss, and elevated fracture risk in certain populations [9,13,33]. Several meta-analyses have confirmed that SSRI users face higher odds of sustaining any fracture than nonusers, with effects observed across hip, vertebral, and appendicular sites [33-35]. However, existing literature has focused predominantly on fracture incidence rather than fracture healing or fixation outcomes, leaving the perioperative period relatively understudied.

A smaller number of studies have examined the influence of systemic medications on fracture repair biology more broadly. Investigations into perioperative non-steroidal anti-inflammatory drug, corticosteroid, and anticoagulant use have demonstrated that pharmacological exposures surrounding fracture fixation can meaningfully influence union rates and complication profiles -- establishing a precedent for examining other chronically administered medications, including SSRIs, as potential modifiers of fixation outcomes [36-38]. This study extends this line of inquiry to SSRIs, representing one of the first large-scale, propensity-matched analyses to specifically examine perioperative SSRI exposure and postoperative fixation outcomes across multiple anatomical sites and constructs.

Clinical implications

The current evidence generated by this study is insufficient to support formal changes to perioperative SSRI management. Abrupt SSRI discontinuation carries well-characterized risks, including SSRI discontinuation syndrome, depression relapse, and anxiety exacerbation. Any recommendation to withhold SSRIs perioperatively would require a far more robust and consistent evidence basis than is presently available [39,40]. Nevertheless, the significant associations identified in the humeral and ulna shaft ORIF cohorts suggest that perioperative SSRI use may warrant further consideration as a patient-level risk factor in preoperative counseling, particularly in patients undergoing ORIF of anatomically vulnerable long bone segments. Surgeons managing these patients may consider incorporating SSRI use into broader discussions of modifiable and non-modifiable risk factors for nonunion and implant failure while coordinating with prescribing providers regarding the overall risk-benefit profile of continued use in the perioperative window.

Limitations

Several limitations of this study merit consideration. First, the retrospective design and reliance on EHR coding introduce inherent risks of misclassification, as the accuracy of ICD-10-CM and CPT coding is variable across institutions and cannot be independently verified. Second, TriNetX does not have granular data on SSRI type, dosing, or duration of use, precluding any dose-response analysis or agent-specific subgroup comparisons. Third, despite propensity score matching on a broad set of covariates, the possibility of residual confounding by unmeasured variables, including depression severity, functional status, nutritional status, physical activity level, and medication adherence, cannot be excluded. MDD itself, independent of SSRI treatment, has been associated with impaired surgical recovery and may contribute to the observed associations [41]. Fourth, selection bias may have influenced cohort composition, as patients prescribed SSRIs likely differ in systematic ways from those who are not, even after matching. Fifth, the exposure window included SSRI use within one month following the index surgery, which introduces the possibility of protopathic bias. Patients who experience early postoperative complications may develop secondary depression or anxiety, prompting new SSRI initiation, such that the complication may have preceded and caused the exposure rather than the reverse. This limits causal inference, particularly in the ORIF cohorts where significant associations were observed. Future studies should restrict exposure to the preoperative period. Sixth, TriNetX does not capture granular operative details such as plate working length, construct stiffness, screw configuration, or reduction quality, all of which independently influence rates of nonunion and implant failure following ORIF and could not be adjusted for during propensity matching. Variability in surgical technique within the ORIF cohorts, therefore, represents an unmeasured confounder that cannot be excluded as a contributor to the observed associations. Seventh, the current exposure definition cannot distinguish patients on established chronic SSRI maintenance therapy from those with recent initiation, nor does it account for dose, adherence, or whether treatment was continued throughout the postoperative period. This exposure heterogeneity may have diluted or distorted the observed associations. Similarly, the control group's definition of no documented SSRI history may misclassify patients with undocumented use. Eighth, this study analyzed six independent cohorts across four outcome measures without formal correction for multiple comparisons, increasing the probability of type I error. Isolated significant findings, particularly those not replicated across anatomically related cohorts, should therefore be interpreted with appropriate caution. Finally, postoperative hematoma was not independently ascertained as a discrete outcome despite its mechanistic relevance to the infection-related implant failure findings observed in the humeral and ulnar ORIF cohorts. Future studies should capture hematoma incidence directly to better evaluate the proposed platelet dysfunction mechanism.

Future directions

The heterogeneous findings of this study highlight the need for rigorous prospective investigation. Future studies should incorporate granular SSRI data, such as specific medication, dosing regimen, and duration of usage, to enable dose-response analyses and drug-specific comparisons. Prospective cohort studies with standardized outcome definitions and longer follow-up windows are needed to better characterize the temporal relationship between SSRI exposure and fracture healing. Investigation of structured perioperative SSRI washout or dose reduction protocols, evaluated in the context of both orthopedic and psychiatric outcomes, would be a meaningful and clinically translatable area of future study.

## Conclusions

Perioperative SSRI usage is associated with increased nonunion and implant failure in select ORIF cohorts, specifically humeral and ulnar shaft, but not in IMN cohorts. The inconsistency of findings across fixation types limits broad conclusions about true SSRI-fracture-healing associations. The observed associations in the ORIF cohorts, while statistically significant, are likely influenced by residual confounding that matching could not fully eliminate, given the inherent limitations of retrospective database methodology. As such, the study hypothesis that perioperative SSRI use uniformly and adversely affects fracture fixation outcomes was not upheld. These findings, nonetheless, contribute meaningful preliminary data to an underexplored area of perioperative orthopedic research and identify specific clinical contexts, namely cortical long bone ORIF, where the influence of SSRI use on healing outcomes may deserve further investigation. Prospective studies with granular pharmacological data and mechanistic investigations into SSRI effects on fracture repair biology are warranted to clarify the clinical significance of these associations and inform evidence-based perioperative management strategies.

## Appendices

Appendix A

**Table 5:** ICD-10 and CPT codes for ORIF cohort identification

Cohort	CPT code	CPT description	Representative ICD-10 diagnosis codes
Humeral shaft ORIF	24515	Open treatment of humeral shaft fracture with plate/screws, with or without cerclage	S42.301A–B, S42.302A–B, S42.309A–B, S42.311A, S42.312A, S42.319A, S42.321A–B, S42.322A–B, S42.323A–B, S42.324A–B, S42.325A–B, S42.326A, S42.331A–B, S42.332A–B, S42.333A–B, S42.334A–B, S42.335A–B, S42.336A, S42.341A–B, S42.342A–B, S42.343A, S42.344A–B, S42.345A–B, S42.346A–B, S42.351A–B, S42.352A–B, S42.353A–B, S42.354A–B, S42.355A–B, S42.356A–B, S42.361A–B, S42.362A–B, S42.363A–B, S42.364A–B, S42.365A–B, S42.366A, S42.391A–B, S42.392A–B, S42.399A–B
Clavicular shaft ORIF	23515	Open treatment of clavicular fracture, includes internal fixation, when performed	S42.001A–B, S42.002A–B, S42.009A–B, S42.011A–B, S42.012A–B, S42.013A–B, S42.014A–B, S42.015A–B, S42.016A–B, S42.017A–B, S42.018A–B, S42.019A–B, S42.021A–B, S42.022A–B, S42.023A–B, S42.024A–B, S42.025A–B, S42.026A
Ulnar shaft ORIF	25545	Open treatment of ulnar shaft fracture, includes internal fixation, when performed	S52.201A–B, S52.202A–B, S52.209A–B, S52.211A–B, S52.212A–B, S52.219A–B, S52.221A–B, S52.222A–B, S52.223A–B, S52.224A–B, S52.225A–B, S52.226A, S52.231A–B, S52.232A–B, S52.233A–B, S52.234A–B, S52.235A–B, S52.236A, S52.241A–B, S52.242A–B, S52.243A, S52.244A–B, S52.245A–B, S52.246A–B, S52.251A–B, S52.252A–B, S52.253A–B, S52.254A–B, S52.255A–B, S52.256A–B, S52.261A–B, S52.262A–B, S52.263A–B, S52.264A–B, S52.265A–B, S52.266A

Appendix B 

**Table 6:** ICD-10 and CPT codes for IMN cohort identification

Cohort	CPT code	CPT description	Representative ICD-10 diagnosis codes
Humeral shaft IMN	24516	Treatment of humeral shaft fracture, with insertion of intramedullary implant, with or without cerclage and/or locking screws	S42.301A–B, S42.302A–B, S42.309A–B, S42.311A, S42.312A, S42.319A, S42.321A–B, S42.322A–B, S42.323A–B, S42.324A–B, S42.325A–B, S42.326A, S42.331A–B, S42.332A–B, S42.333A–B, S42.334A–B, S42.335A–B, S42.336A, S42.341A–B, S42.342A–B, S42.343A, S42.344A–B, S42.345A–B, S42.346A–B, S42.351A–B, S42.352A–B, S42.353A–B, S42.354A–B, S42.355A–B, S42.356A–B, S42.361A–B, S42.362A–B, S42.363A–B, S42.364A–B, S42.365A–B, S42.366A, S42.391A–B, S42.392A–B, S42.399A–B
Femoral shaft IMN	27506	Open treatment of femoral shaft fracture, with or without external fixation, with insertion of intramedullary implant, with or without cerclage and/or locking screws	S72.301A–B, S72.302A–B, S72.309A–B, S72.321A–B, S72.322A–B, S72.323A–B, S72.324A–B, S72.325A–B, S72.326A, S72.331A–B, S72.332A–B, S72.333A–B, S72.334A–B, S72.335A–B, S72.336A, S72.341A–B, S72.342A–B, S72.343A–B, S72.344A–B, S72.345A–B, S72.346A, S72.351A–B, S72.352A–B, S72.353A–B, S72.354A–B, S72.355A–B, S72.356A, S72.361A–B, S72.362A–B, S72.363A–B, S72.364A–B, S72.365A–B, S72.366A, S72.391A–B, S72.392A–B, S72.399A–B
Tibial shaft IMN	27759	Treatment of tibial shaft fracture (with or without fibular fracture) by intramedullary implant, with or without interlocking screws and/or cerclage	S82.201A–B, S82.202A–B, S82.209A–B, S82.221A–B, S82.222A–B, S82.223A–B, S82.224A–B, S82.225A–B, S82.226A, S82.231A–B, S82.232A–B, S82.233A–B, S82.234A–B, S82.235A–B, S82.236A, S82.241A–B, S82.242A–B, S82.243A, S82.244A–B, S82.245A–B, S82.246A–B, S82.251A–B, S82.252A–B, S82.253A–B, S82.254A–B, S82.255A–B, S82.256A–B, S82.261A–B, S82.262A–B, S82.263A–B, S82.264A–B, S82.265A–B, S82.266A

Appendix C 

**Table 7:** RxNorm codes for SSRI exposure classification Note: SSRI exposure was defined as any documented use of the above medications within one month before or after the index surgery. RxNorm concept IDs (CUIs) were used as the medication identifiers within the TriNetX query builder.

SSRI medication	RxNorm concept ID (CUI)
Citalopram	2556
Escitalopram	321988
Fluoxetine	4493
Paroxetine	32937
Sertraline	36437

Appendix D

**Table 8:** ICD-10 codes for outcome ascertainment (assessed at one year post-index surgery) Note: ICD-10 codes ending in A denote initial encounters; S denotes sequela. Codes were applied as inclusion criteria within the TriNetX query builder. All codes reflect ICD-10-CM conventions. Outcome codes were searched in the period from index surgery through one-year follow-up.

Outcome	ICD-10 code(s)	Description
Nonunion	M84.80XA–S, M84.81XA–S, M84.82XA–S, M84.83XA–S, M84.84XA–S, M84.85XA–S, M84.86XA–S, M84.87XA–S, M84.88X; M96.0	Delayed healing and nonunion of fracture (M84.8–); pseudarthrosis after fusion or arthrodesis (M96.0)
Infection-related implant failure	T84.50XA–S, T84.51XA–S, T84.52XA–S, T84.53XA–S, T84.54XA–S, T84.59XA–S, T84.60XA–S, T84.61XA–S, T84.62XA–S, T84.63XA–S, T84.69XA–S	Infection and inflammatory reaction due to internal fixation devices (T84.5–); infection due to other internal orthopedic prosthetic devices (T84.6–)
Mechanical implant failure	T84.190A–S, T84.191A–S, T84.192A–S, T84.193A–S, T84.194A–S, T84.195A–S, T84.196A–S, T84.199A–S, T84.290A–S, T84.291A–S, T84.292A–S, T84.293A–S, T84.296A–S, T84.390A–S, T84.391A–S, T84.392A–S, T84.393A–S, T84.394A–S, T84.398A–S, T84.490A–S, T84.498A–S	Mechanical complication of internal fixation devices of bones (T84.1–); mechanical complication of other internal fixation devices (T84.2–, T84.3–, T84.4–): breakage, displacement, loosening, obstruction, perforation, protrusion
All-cause mortality	Z00–Z99 (vital status field); documented death within TriNetX EHR	All-cause mortality ascertained via TriNetX vital status and linked EHR mortality data; no specific ICD-10 code applied
